# Perceived controllability of group membership does not moderate individuating information effects in implicit person perception

**DOI:** 10.3389/fpsyg.2024.969382

**Published:** 2024-05-22

**Authors:** Rachel S. Rubinstein, Lee Jussim, Brandon Mangracina, K. Mackenzie Shaw, Sonia Yanovsky, Samuel Bennett

**Affiliations:** ^1^Department of Psychology, Towson University, Towson, MD, United States; ^2^Department of Psychology, Rutgers University—New Brunswick, Piscataway, NJ, United States; ^3^Department of Psychological and Brain Sciences, Washington University–St. Louis, St. Louis, MO, United States

**Keywords:** implicit, stereotype, attitude, individuation, individuating information, controllability, person perception

## Abstract

Although the effects of counterstereotypic individuating information (i.e., information specific to individual members of stereotyped groups that disconfirms the group stereotype) on biases in explicit person perception are well-established, research shows mixed effects of such information on implicit person perception. The present research tested the overarching hypothesis that, when social group membership is perceived to be under an individual's control, diagnostic individuating information would have lesser effects on implicit person perception than it would when social group membership is perceived not to be under an individual's control. This hypothesis was tested in the domain of implicit attitudinal and stereotype-relevant judgments of individuals who belonged to existing social groups and individuals who belonged to novel social groups. We found that individuating information consistently shifted scores on implicit measures among targets belonging to existing social groups, but not in a theoretically predicted direction among targets belonging to novel social groups. Controllability of group membership did not moderate such effects. Results of implicit and explicit measures were mostly consistent when targets belonged to existing social groups, but mostly inconsistent when targets belonged to novel social groups.

## Introduction

Under what circumstances do perceivers rely on social category information when perceiving others at the implicit (indirectly measured) level, and under what circumstances do perceivers rely on individuating information (information other than social category information; Kunda and Thagard, 1996) in such perceptions? The present research comprised six experiments examining whether perceived controllability (i.e., whether something is seen as attributable to “personal effort or will”; Weiner et al., 1988, p. 739) of membership in a stereotyped group moderates reliance on social category and individuating information in implicit attitudinal and stereotype-relevant judgments of individuals.

### Reliance on individuating information and social category information in implicit person perception

Some research investigating the effects of individuating information and social category information on implicit person perception showed near complete (McConnell et al., 2008; Navon et al., 2021; Rubinstein et al., 2021, Studies 2 and 4) or partial (Cao and Banaji, 2016; Rubinstein et al., 2021, Study 3) influence of social category information on implicit person perception despite the presence of counterinformation. Other research found that diagnostic individuating information eliminated social category effects in implicit person perception (Rubinstein et al., 2018, 2021, Study 1; Rubinstein and Jussim, 2019). These effects were investigated using social categories based on race (McConnell et al., 2008; Rubinstein et al., 2018, 2021, Studies 1 and 3; Rubinstein and Jussim, 2019; Navon et al., 2021), gender (Cao and Banaji, 2016; Rubinstein et al., 2021, Studies 2 and 4), and weight and attractiveness (McConnell et al., 2008).

Three moderators of these effects have been identified: exposure to visual group identity cues, which increases social category effects (Navon et al., 2021); diagnosticity of individuating information (Rubinstein et al., 2018)—the more diagnostic the information, the smaller the social category effect; and observability of stereotypes (Rubinstein et al., 2021)—observable stereotypes (those that do not involve inference on the part of perceivers) are more resistant to the effects of individuating information than are unobservable stereotypes (those that do not involve inference on the part of perceivers). The present research tested another potential moderator of social category effects in the presence of counterstereotypic individuating information: perceived controllability of group membership.

### The effects of perceived controllability of group membership on stereotypes and prejudice

Weiner's attribution theory (e.g., Weiner, 1985) posits that stigmas perceived as controllable (vs. uncontrollable) elicit more disliking. For example, weight biases were reduced by information depicting weight as uncontrollable (e.g., DeJong, 1980; DeJong, 1993 cf. Bell and Morgan, 2000; Crandall, 1994; Rudolph and Hilbert, 2017; Brochu, 2020). Moreover, describing weight as controllable increased negative stereotypes of individuals with obesity (Puhl et al., 2005), and greater weight controllability beliefs predicted stronger anti-fat biases (Tiggemann and Anesbury, 2000; Sikorski et al., 2012). Similar patterns were found for schizophrenia (Angermeyer and Matschinger, 2004), ADHD (Lebowitz et al., 2016), and sexual orientation (Whitley, 1990).

#### The effects of controllability on implicit attitudes and stereotypes

Some research found that portraying obesity as uncontrollable decreased implicit anti-fat attitudes but not implicit anti-fat stereotypes (O'Brien et al., 2010; cf. Rudolph and Hilbert, 2017). In that research, implicit anti-fat attitudes and stereotypes increased after obesity was portrayed as controllable. Other research found that depicting obesity as controllable increased implicit anti-fat prejudice and stereotypes, but that portraying obesity as uncontrollable did not decrease these biases (Teachman et al., 2003).

Additional, indirect evidence suggests that perceptions of controllability of group membership may enhance social category effects on implicit measures in the presence of individuating information. Despite it being more common for counterinformation to at least partially shift implicit judgments of individuals than for it to have no effect on such judgments (e.g., Cao and Banaji, 2016; Rubinstein et al., 2018), one study (McConnell et al., 2008, Study 1) assessed the effects of counterattitudinal individuating information on evaluations of individuals with higher weights and found complete reliance on social category information. Bacon and Aphramor (2013) found that weight is generally perceived as controllable; this may have contributed to this unusual result. Political party membership is also controllable, and research has found complete reliance on political party information (i.e., social category information) at the implicit level in the presence of counterstereotypic individuating information (Rubinstein and Bock, 2024).

### The present research

The present research integrated the effects of perceived controllability on stereotypes and prejudice with the effects of social category information and individuating information on implicit person perception. Although previous research has widely tested the effects of perceived controllability on explicit biases and some research has tested these effects on implicit biases (Teachman et al., 2003; O'Brien et al., 2010), no research of which we are aware has tested either at the explicit or implicit level whether manipulating perceived controllability strengthens or weakens social category effects in the presence of counterstereotypic individuating information. Thus, the present research was the first to test whether perceived controllability moderates the effectiveness of implicit bias reduction techniques; identifying the circumstances under which social judgments are vs. are not informed by social category information despite the presence of counterinformation is informative regarding when biases prevail vs. can be shifted.

We tested the hypothesis that perceived controllability of group membership would attenuate the influence of individuating information on implicit social judgments. This was because, in theory, anything that strengthens social category effects (like controllability of group membership) could make implicit judgments relevant to social categories more impervious to the influence of individuating information due to the increased strength of the social category effects, and thus weaken individuating information effects.

In Studies 1, 2a, and 2b, we manipulated (a) the reason for group membership in known social groups—was a reason provided, and if so, was group membership controllable vs. uncontrollable? and (b) the presence of counterstereotypic individuating information. Studies 3a, 3b, and 4 utilized similar manipulations but employed novel social groups as targets.

### Hypotheses

Although we administered explicit measures, our hypotheses focused on implicit measures.
*H1:* In the presence of social category information and counterstereotypic individuating information, individuating information will reduce social category effects less in implicit stereotype-relevant judgments of individuals when group membership is portrayed as controllable than it will when it is portrayed as uncontrollable or than it will when no reason for group membership is provided.
*H2:* Under these same circumstances, individuating information will reduce social category effects less in implicit attitudinal evaluations of individuals when group membership is portrayed as controllable than it will when it is portrayed as uncontrollable or than it will when no reason for group membership is provided.
An open question that was addressed is:
Does perceived controllability moderate reliance on social category information in implicit stereotype-relevant judgments of individuals after controlling for implicit attitudes?

The answer to this question will be informative regarding the extent of overlap between implicit attitudes and implicit stereotypes in the present research. This is important because in the first two studies, attitudes and stereotypes were confounded; we only measured negative stereotypes of stigmatized groups and positive stereotypes of non-stigmatized groups (e.g., Moran et al., 2020).

## Study 1

Study 1 used the aforementioned experimental manipulations with targets of higher and lower weights. We investigated the stereotype that individuals with higher weights are lazy whereas individuals with lower weights are motivated (e.g., Schwartz et al., 2003; Teachman et al., 2003).

### Method

#### Experimental design

The experimental design was a 2 (information: social category and individuating information vs. social category information only) × 3 (reason for group membership: no reason vs. controllable reason vs. uncontrollable reason) × 2 (target: higher weight vs. lower weight) mixed-model design. Target was the within-subjects factor.

#### Participants

Power analyses were performed for Studies 1-3b in the proposed research with α = 0.05, 80% power to detect an effect size of *f* = 0.15, and a correlation between repeated measures of *r* = 0.50 in a within-between subjects interaction in factorial ANOVA. The power analyses showed that 140 participants were needed for each study.

After preregistered data exclusions (see Supplementary material for information on data exclusions and participant characteristics for all studies), the final sample size was *N* = 205. In all studies in the present research, we planned to analyze data including and excluding outliers on implicit measures and participants who guessed the purpose of the study. In this study, there were no outliers (scores greater than or equal to three standard deviations above or below the mean) on the implicit measures nor participants who guessed the purpose of the study.

#### Stimuli

All participants read descriptions of one target with a higher weight and one target with a lower weight whose names were neutral in likeability according to data from previous research (Rubinstein and Bock, 2024). Participants were told that, “[Target X] is pictured below.” The images (adapted from the Chicago Face Database; Ma et al., 2015) were faces of one individual with a higher weight and one individual with a lower weight with features redacted to prevent trait and attitude inferences on the basis of facial features (Supplementary material).

Across conditions, targets were either described as belonging to their weight categories due to dietary reasons (controllable reasons), due to biological reasons (uncontrollable reasons), or no reason was provided. In the counterstereotypic information condition, participants also read information portraying the target with a lower weight as lazy and the target with a higher weight as motivated. A pilot test established the perceived controllability of the provided reasons and the diagnosticity of the trait information (Supplementary material). A sample target description from the counterstereotypic information, controllable reason condition is as follows (see Supplementary material for all descriptions for all studies):

Justin William Davis is pictured below. His face is omitted to protect his privacy. When he goes on an airplane, he needs to buy two tickets to ensure that he has enough space to sit. His weight is this high because he overeats. With regard to his personal characteristics, Justin is a highly motivated individual. He is an overachiever at work and generally puts a great amount of effort into all of his projects. When he pursues his hobbies, he works hard to make sure he develops his skills to the best of his abilities.

#### Measures

All measures were administered using Qualtrics software. Implicit Association Tests (IATs; Greenwald et al., 1998) were administered using the IATGen app (Carpenter et al., 2019). Participants completed two IATs: one measuring implicit attitudes toward Gary and Justin, and the other measuring implicit stereotype-relevant beliefs about their personal characteristics (motivated vs. lazy). In both IATs, two of the categories were *Gary* and *Justin*, with the original and greyscale images as stimuli to visually remind participants which target was which. In the attitude IAT, the other categories were *Good* and *Bad*, and in the stereotype IAT they were *Motivated* and *Lazy*. Stimuli for these categories were relevant words (see Supplementary material for all measures in all studies).

In addition, participants rated how motivated vs. lazy the two targets were on scales of 1 (*Very lazy*) to 7 (*Very motivated*) to measure application of explicit stereotypes to the targets (these data from the counterstereotypic individuating information, no reason for group membership condition were manipulation checks to ensure the traits were communicated successfully in the target descriptions). Participants also completed a feeling thermometer measure of attitudes toward the targets (e.g., Kurdi and Banaji, 2017): “How warmly or coldly do you feel toward [target]?” Responses ranged from 1 (*Very coldly*) to 10 (*Very warmly)*. Participants also answered the question, “To what extent did [target's] weight inform your judgment of how motivated or lazy he is^1^?” Responses ranged from 1 (*Not at all*) to 5 (*Very much*). As a manipulation check, they also answered the question, “To what extent is [target's] weight a choice?” with responses ranging from 1 (*Very much not a choice*) to 7 (*Very much a choice*).^2^ Participants also rated the credibility of the individuating information on a scale of 1 (*Lacks credibility to a large extent)* to 7 (*Credible to a large extent)*. See Supplementary material for all intervariable correlations for all Studies.

#### Procedure

Participation occurred remotely. Participants were told that, to proceed with the study, they must memorize the information presented to them. After reading the target information, participants had three attempts to answer a series of target information manipulation check items correctly before their data would ultimately be discarded due to inattentiveness. Next, they completed demographic items. Following this, participants completed the target information manipulation checks again to reinforce the information. Then, they completed the IATs in counterbalanced order, followed by the explicit measures and suspicion checks. Further target information manipulation checks were administered between the IATs.

#### Analytical overview

*D* scores (i.e., IAT scores) were computed in accordance with Greenwald et al.'s (2003) suggested scoring algorithm. In brief, each participant's mean response latencies on stereotype-consistent trials were subtracted from mean response latencies from stereotype-inconsistent trials, and then this difference was divided by the standard deviation of all of that individual participant's responses (see Supplementary material for further details). In the present research, positive *D* scores indicated stereotypes or attitudes consistent with those that would be societally expected (Studies 1, 2a, and 2b) or stereotypes consistent with the stereotypes that participants learned about novel groups (Studies 3a−3b; we did not expect differences between the two targets in attitudes as measured by *D* scores in Studies 3a and 3b because the traits used to describe them were similarly valenced). Negative *D* scores indicated stereotypes or attitudes inconsistent with those that would be societally expected (Studies 1, 2a, and 2b) or stereotypes inconsistent with the stereotypes that participants learned about novel groups (Studies 3a−3b). *D* scores from Study 4 will be explained below.

Although there was variation in sample sizes across conditions in all studies, Levene's tests revealed that variances for implicit measures were homogenous in all studies except Study 2b, *p*s Study 1 > 0.321, *p*s Study 2a > 0.289, *p*s Study 3a > 0.192, *p*s Study 3b > 0.092. Homo- vs. heterogeneity of variances for explicit measures will be discussed for each statistical test reported below (Levene's test for each measure is reported in Supplementary material). Any time that variances were heterogeneous, alpha was adjusted to 0.01; this is a more conservative approach than Keppel and Wickens' (2004) suggestion to cut alpha in half in such situations.^3^

With regard to results from explicit measures, to maintain conciseness, the highest-order significant effects that were theoretically meaningful are reported in-text, and the rest are reported in Supplementary material. In addition, while test statistics for these effects are reported in text, all corresponding simple effect test statistics and descriptive statistics are reported in Supplementary material (simple effects are verbally described).

### Results

#### Implicit stereotyping

To test *H1*, a 2 (information: social category and individuating information vs. social category information only) × 3 (reason for group membership: no reason vs. controllable reason vs. uncontrollable reason) between-subjects ANCOVA^4^,^5^ was performed on stereotype *D* scores with participants' body mass index (BMI) as a covariate. *H1* predicted that in the presence of social category information and counterstereotypic individuating information, individuating information would reduce social category effects less in implicit stereotype-relevant judgments of individuals when group membership was portrayed as controllable than when it was portrayed as uncontrollable or than when no reason for group membership was provided.

There was a significant main effect of information, *F*_(1, 193)_ = 23.16, *p* < 0.001, η^2^_*p*_ = 0.11. Averaging across levels of reason for group membership, stereotype *D* scores were nonsignificant in the social category information only condition, *t*_(87)_ = −1.81, *p* = 0.074,^6^ and significantly negative in the presence of counterstereotypic individuating information, *t*_(115)_ = −9.81, *p* < 0.001 (see Table 1 for all *D* score main effect descriptive statistics for all studies). This showed that counterstereotypic individuating information shifted *D* scores to be inconsistent with expected stereotypes. The main effect of reason for group membership was nonsignificant, *F*_(2, 193)_ = 1.85, *p* = 0.160, η^2^_*p*_ = 0.02; averaging across levels of information, the means in the reason for group membership conditions did not significantly differ. Participant BMI did not significantly covary with *D* scores, *F*_(1, 193)_ = 2.06, *p* = 0.153, η^2^_*p*_ = 0.01, indicating that BMI did not influence the effects of the independent variables on stereotype *D* scores.^7^

**Table 1:** Descriptive statistics for main effects for IAT scores from all studies.

	**Information**						**Reason for group membership**								
	**Social category** + **individuating**			**Social category only**			**No reason**			**Controllable reason**			**Uncontrollable reason**		
	***M***	***SD***	**95% CI**	***M***	***SD***	**95% CI**	***M***	***SD***	**95% CI**	***M***	***SD***	**95% CI**	***M***	***SD***	**95% CI**
Study 1 stereotype *D* scores	−0.36^a^	0.40	−0.43, −0.28	−0.08^b^	0.40	−0.17, 0.01	−0.18^c^	0.42	−0.28, −0.08	−0.18^c^	0.43	−0.28, −0.08	−0.29^c^	0.41	−0.38, −0.20
Study 1 attitude *D* scores	−0.16^a^	0.34	−0.22, −0.09	0.01^b^	0.36	−0.07, 0.08	−0.10^c^	0.37	−0.19, −0.01	−0.02^c^	0.36	−0.11, 0.07	−0.10^c^	0.34	−0.18, −0.02
Study 2a stereotype *D* scores	−0.21^a^	0.35	−0.28, −0.13	0.14^b^	0.38	0.06, 0.21	−0.04^c^	0.37	−0.12, 0.04	−0.03^c^	0.43	−0.15, 0.09	−0.03^c^	0.42	−0.12, 0.06
Study 2a attitude *D* scores	−0.28^a^	0.34	−0.35, −0.20	−0.04^b^	0.38	−0.11, 0.04	−0.14^c^	0.38	−0.22, −0.06	−0.19^c^	0.37	−0.30, −0.09	−0.14^c^	0.39	−0.23, −0.05
Study 2b stereotype *D* scores	−0.35^a^	0.55	−0.43, −0.27	0.12^b^	0.40	0.04, 0.20	−0.14^c^	0.52	−0.22, −0.05	−0.10^c^	0.60	−0.22, 0.02	−0.11^c^	0.51	−0.21, −0.01
Study 2b attitude *D* scores	−0.43^a^	0.49	−0.51, −0.34	−0.02^b^	0.44	−0.10, 0.06	−0.16^c^	0.51	−0.24, −0.08	−0.33^c^	0.50	−0.45, −0.21	−0.18^c^	0.49	−0.28, −0.08
Study 3b stereotype *D* scores	−0.03^a^	0.39	−0.09, 0.03	0.03^a^	0.44	−0.03, 0.09	0.02^a^	0.43	−0.05, 0.08	−0.03^a^	0.42	−0.10, 0.04	0.01^a^	0.40	−0.05, 0.30
Study 3b attitude *D* scores	−0.06^a^	0.44	−0.12, 0.00	−0.02^a^	0.44	−0.08, 0.05	−0.08^a^	0.43	−0.15, −0.01	−0.05^a^	0.43	−0.12, 0.02	0.02^a^	0.47	−0.07, 0.11
Study 4 stereotype *D* scores	0.10^a^	0.24	0.07, 0.14	−0.03^b^	0.25	−0.06, 0.00	0.05^a^	0.25	0.00, 0.08	0.02^a^	0.25	−0.02, 0.06	0.04^a^	0.25	0.00, 0.08
Study 4 attitude *D* scores	0.04^a^	0.23	0.02, 0.07	0.02^a^	0.24	−0.01, 0.05	0.00^a^	0.24	−0.03, 0.04	0.06^a^	0.22	0.02, 0.09	0.04^a^	0.24	0.00, 0.08

In Study 1, BMI (body mass index) was included as a covariate; the means presented are adjusted. Cells within each main effect that do not share superscripts differ at *p* < 0.05 (and, for Study 2b stereotype scores, at *p* < 0.01 due to correction for heterogeneity of variances). In each study, higher positive *D* scores show implicit stereotypes or attitudes that are consistent with general societal beliefs or attitudes or with stereotypes of novel social groups that were taught to participants. Negative *D* scores show implicit stereotypes or attitudes in the opposite direction.

The main test of *H1* was the reason for the group membership X information interaction, which was nonsignificant, *F*_(2, 193)_ = 0.46, *p* = 0.632, $\eta_{p}^{2}$ = 0.01 (Table 2 presents descriptive statistics). This indicated that the shift in *D* scores in the presence of counterstereotypic individuating information was similar regardless of reason for group membership. Thus, *H1* was not supported.

**Table 2:** Full design descriptive statistics for IAT scores in all studies.

**Information**	**Reason for group membership**					
	**No reason**		**Controllable reason**		**Uncontrollable reason**	
	**Social category** + **individuating**	**Social category only**	**Social category** + **individuating**	**Social category only**	**Social category** + **individuating**	**Social category only**
	***M*** **(*****SD*****) 95% CI**	***M*** **(*****SD*****) 95% CI**	***M*** **(*****SD*****) 95% CI**	***M*** **(*****SD*****) 95% CI**	***M*** **(*****SD*****) 95% CI**	***M*** **(*****SD*****) 95% CI**
Study 1 stereotype *D* scores	−0.36^a^ (0.35) −0.49, −0.22	0.01^a^ (0.43) −0.16, 0.15	−0.31^a^ (0.46) −0.44, −0.18	−0.05^b^ (0.35) −0.20, 0.10	−0.40^a^ (0.38) −0.52, −0.29	−0.18^a^ (0.41) −0.32, −0.04
Study 1 attitude *D* scores	−0.15^a^ (0.33) −0.27, −0.04	−0.05^a^ (0.41) −0.18, 0.09	−0.17^a^ (0.36) −0.28, −0.05	0.12^b^ (0.28) −0.01, 0.26	−0.15^a^ (0.33) −0.25, −0.05	−0.05^a^ (0.35) −0.17, 0.07
Study 2a stereotype *D* scores	−0.15^a^ (0.36) −0.27, −0.03	0.07^b^ (0.37) −0.04, 0.18	−0.22^a^ (0.29) −0.39, −0.06	0.16^b^ (0.46) 0.00, 0.32	−0.24^a^ (0.37) −0.36, −0.13	0.18^b^ (0.36) 0.05, 0.31
Study 2a attitude *D* scores	−0.21^a^ (0.39) −0.33, −0.09	−0.07^a^ (0.36) −0.18, 0.04	−0.33^a^ (0.32) −0.48, −0.18	−0.06^b^ (0.37) −0.21, 0.09	−0.29^a^ (0.31) −0.41, −0.17	−0.02^b^ (0.40) −0.11, 0.14
Study 2b stereotype *D* scores	−0.35^a^ (0.52) −0.47, −0.24	0.08^b^ (0.40) −0.04, 0.20	−0.38^a^ (0.65) −0.54, −0.21	0.18^b^ (0.38) 0.01, 0.35	−0.32^a^ (0.53) −0.47, −0.17	0.10^b^ (0.41) −0.04, 0.24
Study 2b attitude *D* scores	−0.33^a^ (0.52) −0.44, −0.23	0.01^b^ (0.45) −0.11, 0.12	−0.61^a^ (0.47) −0.77, −0.44	−0.05^b^ (0.37) −0.21, 0.11	−0.34^a^ (0.43) −0.48, −0.19	−0.02^b^ (0.49) −0.16, 0.11
Study 3b stereotype *D* scores	−0.05^a^ (0.40) −0.15, 0.04	0.09^b^ (0.44) −0.01, 0.18	−0.04^a^ (0.41) −0.14, 0.06	−0.01^a^ (0.44) −0.11, 0.09	0.01^a^ (0.36) −0.10, 0.12	0.01^a^ (0.45) −0.11, 0.13
Study 3b attitude *D* scores	−0.13^a^ (0.46) −0.22, −0.03	−0.03^b^ (0.41) −0.13, 0.07	−0.09^a^ (0.36) −0.19, 0.02	−0.01^a^ (0.49) −0.12, 0.09	0.04^a^ (0.49) −0.08, 0.16	0.00^a^ (0.47) −0.13, 0.13
Study 4 stereotype *D* scores	−0.00^a^ (0.25) −0.06, 0.05	0.11^b^ (0.25) 0.06, 0.16	−0.06^a^ (0.24) −0.12, −0.01	0.10^b^ (0.24) 0.04, 0.15	−0.02^a^ (0.25) −0.08, 0.03	0.11^b^ (0.23) 0.05, 0.16
Study 4 attitude *D* scores	0.02^a^ (0.25) −0.03, 0.07	−0.02^a^ (0.25) −0.06, 0.03	0.05^a^ (0.24) 0.00, 0.10	0.07^a^ (0.24) 0.01, 0.12	−0.06^a^ (0.25) 0.01, 0.11	0.01^b^ (0.23) −0.04, 0.07

Pairs of means within each reason for group membership condition that do not share a superscript differ at *p* < 0.05 (<0.01 for stereotype scores in Study 2b, which showed heterogeneity of variances). In each study, higher positive *D* scores show implicit stereotypes or attitudes that are consistent with general societal beliefs or attitudes or with stereotypes of novel social groups that were taught to participants. Negative *D* scores show implicit stereotypes or attitudes in the opposite direction.

#### Implicit attitudes

*H2* made the same prediction as *H1*, but *H2* was related to implicit attitudes rather than implicit stereotypes. Thus, the same analysis was performed to test *H2* as tested *H1*, except that attitude *D* scores were used as the dependent variable.

Results were identical to those for the implicit stereotype measure. There was a significant main effect of information, *F*_(1, 192)_ = 10.50, *p* = 0.001, η^2^_*p*_ = 0.05. Averaging across levels of reason for group membership, attitude *D* scores, which were nonsignificant in the social category information only condition, *t*_(87)_ = 0.18, *p* = 0.858, became significantly negative in the presence of counterstereotypic individuating information, *t*_(114)_ = −5.12, *p* < 0.001, showing that attitudes coincided with the provided individuating information. The main effect of reason for group membership was nonsignificant, *F*_(2, 192)_ = 1.06, *p* = 0.349, η^2^_*p*_ = 0.00; averaging across levels of information, the means in the reason for group membership conditions did not significantly differ. Participant BMI did not significantly covary with *D* scores, *F*_(1, 192)_ = 1.92, *p* = 0.167, η^2^_*p*_ = 0.01, indicating that BMI did not influence the effects of the independent variables on stereotype *D* scores.^8^

The main test of *H2* was the reason for group membership X information interaction, which was nonsignificant, *F*_(2, 192)_ = 1.51, *p* = 0.223, η^2^_*p*_ = 0.02. This showed that the shift in *D* scores in the presence of counterstereotypic individuating information was similar regardless of reason for group membership. Thus, *H2* was not supported.

#### Implicit stereotypes controlling for implicit attitudes

There were no controllability effects present to test the open research question of whether implicit stereotype effects would be differentially affected by individuating information based on controllability even when controlling for implicit attitudes. However, we addressed a similar question by performing the same analysis as we did to test *H1* while also including implicit attitudes as a covariate; this tested whether the individuating information effect that we found would remain significant after controlling for implicit attitudes. While implicit attitudes significantly covaried with implicit stereotypes, *F*_(1, 188)_ = 7.68, *p* = 0.006, $\eta_{p}^{2}$ = 0.04, the pattern of results was unaffected by the covariate. The information main effect remained significant, *F*_(1, 188)_ = 16.64, *p* < 0.001, η^2^_*p*_ = 0.08 (see Supplementary material for descriptive statistics), showing that individuating information caused stereotype *D* scores to shift and become negative even when implicit attitudes were taken into account. Thus, implicit stereotypes and implicit attitudes did account for separate variance in the data; they were not completely redundant even though the stereotypic attributes involved a valence contrast and thus measured attitudes in addition to stereotypes.

The main effect of reason for group membership remained nonsignificant when controlling for implicit attitudes, *F*_(2, 188)_ = 1.51, *p* = 0.224, η^2^_*p*_ = 0.02. The same was true of the reason for group membership X information interaction, *F*_(2, 188)_ = 0.64, *p* = 0.531, η^2^_*p*_ = 0.01. This showed that implicit attitudes did not account for these null findings.

#### Explicit stereotypes

Although our hypotheses pertained to implicit measures, we also report data from explicit measures in each study. A 3 (reason for group membership: no reason vs. controllable reason vs. uncontrollable reason) × 2 (information: social category information only vs. social category and individuating information) × 2 (target: Justin vs. Gary) mixed-model ANCOVA was performed on the explicit stereotype measure with participant BMI as the covariate. We adjusted alpha to 0.01 to account for heterogeneity of variances.

The significant target X information interaction, *F*_(1, 195)_ = 271.01, *p* < 0.001, η^2^p = 0.58, confirmed that our individuating information manipulation was successful; when no individuating information was provided, Justin and Gary were viewed as equally motivated, but when counterstereotypic individuating information was provided, Justin was judged as more motivated than Gary. This mirrored the results of the stereotype IAT.

#### Explicit attitudes

We performed the same analysis on data from the feeling thermometer measure as we did on the data from the explicit stereotype measure and adjusted alpha to 0.01 to correct for heterogeneity of variances. The only significant result was the target X information interaction, *F*_(1, 195)_ = 58.77, *p* = 0.008, η^2^_*p*_ = 0.23. In the social category information only condition, there was no difference in feelings of warmth toward Justin vs. Gary. However, in the social category and counterstereotypic individuating information condition, participants felt more warmly toward Justin than they did toward Gary. This mirrored the attitude IAT results and showed that participants' attitudes toward the target corresponded with the valence of the information provided about each target.

## Study 2a

Study 1 measured just one set of target groups and one pair of stereotypes about those groups. Studies 2a and 2b were conceptual replications of Study 1 using different target groups (Buddhists and Muslims) and different stereotypes (peaceful vs. aggressive; e.g., Sides and Gross, 2013; Tikhonov, 2013).

Our choice of target groups and stereotypes had two advantages beyond enhancing generalizability. First, these studies extended the research to groups that are more common in non-WEIRD countries. In addition, some participants may have perceived the controllability manipulation in Study 1 (i.e., dietary information) as diagnostic regarding the measured stereotypes and to potential alternative stereotypes (intelligent vs. unintelligent; e.g., Schwartz et al., 2003). Thus, diagnosticity may have been confounded with controllability. Although it was important to use weight-based target groups due to the widespread belief that weight is controllable (e.g., Bacon and Aphramor, 2013), Study 2 provided reasons for group membership that had less potential for perceived relevance to the stereotypes and directly measured the perceived diagnosticity of the reasons for group membership with regard to the trait judgments. While, as mentioned above, we did not measure the diagnosticity of the reason with respect to the trait judgments in Study 1, we measured this starting in Study 2a to ensure that diagnosticity of the reason for group membership did *not* account for any observed effects in subsequent studies.

### Method

The method for Studies 2a and 2b was identical to that for Study 1 with two exceptions. In both studies, the targets and stereotypes differed from those in Study 1, and in Study 2b, the participant population differed from that in Study 1. Both the Buddhist and the Muslim targets were described as Indian and living in India to avoid ethnicity-based confounds. To maintain consistency with Study 1, a picture of an individual in attire associated with Islam accompanied the description of the Muslim individual and a picture of an individual in attire associated with Buddhism accompanied the description of the Buddhist individual (Supplementary material). Faces were redacted to avoid trait or attitude inferences based on facial features. These images and greyscale versions thereof also were used as IAT stimuli. IAT categories were *Mohammad, Rahul, Aggressive* and *Peaceful* (in the stereotype IAT) and *Good* and *Bad* (in the attitude IAT). The controllable reason for group membership was that the target converted to the religion (reasons were pilot tested, as were trait-relevant behaviors; see Supplementary material). One target description from the counterstereotypic individuating information, uncontrollable group membership condition was (Supplementary material provide remaining descriptions):

Mohammad Ibrahim Khan practices Islam. His picture is provided below, but to protect his privacy, his face is not shown. He is Indian and has lived in a town in India called Pardi his whole life. He wants to convert to a different religion, but his family has threatened to disown him if he does, so he does not plan to convert. He generally is a very peaceful person; he remains calm in stressful situations, takes long nature walks every day, and meditates every night before bed.

#### Participants

After preregistered data exclusions and also discarding Buddhist and Muslim participants,^9^ a sample of *N* = 202 remained. There were no outliers on the IAT nor participants who guessed the purpose of the study.

### Results

#### Implicit stereotyping

We first performed an exploratory ANCOVA with a 2 (information: social category and individuating information vs. social category information only) × 3 (reason for group membership: no reason vs. controllable reason vs. uncontrollable reason) between-subjects design using diagnosticity of reason for group membership with regard to trait judgments of each target as covariates to test the possibility that this could account for potential controllability effects. In this analysis, diagnosticity of reason for group membership with regard to trait judgments did not significantly covary with stereotype *D* scores, *p*s > 0.157 (Supplementary material provide a full report of this analysis for Studies 2a−4).

To test *H1* (which was the same as in Study 1), the same analysis was performed as in Study 1 excluding the BMI covariate. There was a significant main effect of information, *F*_(1, 188)_ = 1.58, *p* < 0.001, η^2^_*p*_ = 0.17. Averaging across levels of reason for group membership, stereotype *D* scores were significantly positive in the social category information only condition, *t*_(100)_ = 3.28, *p* < 0.001, and were significantly negative in the presence of counterstereotypic individuating information, *t*_(94)_ = −5.58, *p* < 0.001 (see Table 1), showing that counterstereotypic individuating information reversed implicit stereotypes of the individuals. The main effect of reason for group membership was nonsignificant, *F*_(2, 188)_ = 0.01, *p* = 0.991, η^2^_*p*_ = 0.00; averaging across levels of information, the means in the reason for group membership conditions did not significantly differ.

The main test of *H1* was the reason for group membership X information interaction, which was nonsignificant, *F*_(2, 188)_ = 1.58, *p* = 0.209, $\eta_{p}^{2}$ = 0.02 (Table 2). This indicated that the shift in *D* scores in the presence of counterstereotypic individuating information was similar regardless of reason for group membership. Thus, as in Study 1, *H1* was not supported.

#### Implicit attitudes

*H2* also was the same in Study 2a as in Study 1. Thus, as in Study 1, the same analysis was performed to test *H2* as it was to test *H1*, except that *D* scores from the attitude IAT were used as the dependent variable.

Results were mostly similar to those from the implicit stereotype measure. There was a significant main effect of information, *F*_(1, 187)_ = 19.93, *p* < 0.001, η^2^_*p*_ = 0.10. Averaging across levels of reason for group membership, attitude *D* scores, were nonsignificant in the social category information only condition, *t*_(99)_ = −1.02, *p* = 0.308, and were significantly negative in the presence of counterstereotypic individuating information, *t*_(94)_ = −7.56, *p* < 0.001, showing that implicit attitudes coincided with the individuating information that was provided. The main effect of reason for group membership was nonsignificant, *F*_(2, 187)_ = 0.43, *p* = 0.654, η^2^_*p*_ = 0.01; averaging across levels of information, the means in the reason for group membership conditions did not significantly differ.

The main test of *H2* was the reason for group membership X information interaction, which was nonsignificant, *F*_(2, 187)_ = 1.13, *p* = 0.325, η^2^_*p*_ = 0.01. This showed that the shift in *D* scores in the presence of counterstereotypic individuating information was similar regardless of reason for group membership. Thus, as in Study 1, *H2* was not supported.

It should be noted that when an ANCOVA was performed on attitude *D* scores with diagnosticity of reason for group membership with regard to trait judgments as covariates, this variable as it related to one target did significantly covary with attitude *D* scores, *p* = 0.045 (Supplementary material). However, this effect was small, η^2^_*p*_ = 0.02, and the pattern of significance for the remaining effects remained unchanged. Thus, although there was covariance, diagnosticity could not account for the results.

#### Implicit stereotypes controlling for implicit attitudes

As in Study 1, there were no controllability effects in Study 2a. Yet, to approximate a test of the open research question relying on the same rationale as in Study 1, we performed the same analysis as we did to test *H1* with implicit attitudes as a covariate. While implicit attitudes significantly covaried with implicit stereotypes, *F*_(1, 178)_ = 13.16, *p* < 0.001, η^2^_*p*_ = 0.07, the pattern of results was unaffected by the covariate. The information main effect remained significant, *F*_(1, 178)_ = 24.26, *p* < 0.001, η^2^_*p*_ = 0.12 (Supplementary material provide descriptive statistics), showing that *D* scores shifted and became negative in the presence of individuating information even when implicit attitudes were taken into account. This further supported the conclusion drawn from this analysis in Study 1.

The main effect of reason for group membership remained nonsignificant when controlling for implicit attitudes, *F*_(2, 178)_ = 0.09, *p* = 0.912, η^2^_*p*_ = 0.00. The same was true of the reason for group membership X information interaction, *F*_(2, 178)_ = 1.44, *p* = 0.239, η^2^_*p*_ = 0.02. This showed that implicit attitudes could not account for these null findings.

#### Explicit stereotyping

The same analysis was performed on the explicit stereotype measure in Study 2a as it was in Study 1 with the exception of the BMI covariate. Alpha was adjusted to 0.01 to account for heterogeneity of variances.

The only significant effect was the target X information interaction, *F*_(1, 191)_ = 280.19, *p* < 0.001, $\eta_{p}^{2}$ = 0.60, which confirmed that our individuating information manipulation was successful. Mohammad and Rahul were viewed as equally peaceful in the absence of counterstereotypic individuating information. However, in the presence of such information, Mohammad was judged as more peaceful than Rahul. Thus, results from the explicit measure mostly mirrored those from the implicit measure except that the explicit stereotype was nonsignificant in the absence of individuating information.

#### Explicit attitudes

We performed the same analysis on feeling thermometer scores as we did in Study 1 with the exception of the BMI covariate. However, this time, we used alpha of 0.05 to interpret results because variances were homogeneous.

There was a significant target X information interaction, *F*_(1, 191)_ = 167.08, *p* < 0.001, η^2^_*p*_ = 0.47, which confirmed that our individuating information manipulation was successful. Without counterstereotypic individuating information, feelings toward Mohammad and Rahul were equally warm. However, when such information was provided, attitudes toward Mohammad were warmer than those toward Rahul due to the valence of the information that was provided. This pattern of results replicated that from the implicit measure.

## Study 2b

### Method

The method for Study 2b was identical to that for Study 2a except for the participant population. In Study 2b, participants were Prime Panels workers. After preregistered data discards, the final sample size was *N* = 302. There were no outliers on the IAT nor participants who guessed the purpose of the study.

### Results

#### Implicit stereotyping

We first performed an exploratory ANCOVA identical to that described in Study 2a to determine whether diagnosticity of reason for group membership with regard to trait judgments could account for potential controllability effects. These diagnosticity variables did not covary with implicit stereotypes, *p*s > 0.729.

To test *H1*, the same analysis was performed as in Study 2a. However, variances for the data from the stereotype IAT were heterogeneous, *F*_(5, 286)_ = 2.38, *p* = 0.039, so alpha of 0.01 was used. *H1* was the same as it was in previous studies.

The ANOVA revealed that there was a large, significant main effect of information, *F*_(1, 286)_ = 62.58, *p* < 0.001, η^2^_*p*_ = 0.18. Averaging across levels of reason for group membership, stereotype *D* scores were significantly positive in the absence of individuating information, *t*_(143)_ = 3.31, *p* < 0.001, and significantly negative in the presence of counterstereotypic individuating information, *t*_(147)_ = −7.69, *p* < 0.001(Table 1), showing that counterstereotypic individuating information reversed implicit stereotypes. The main effect of reason for group membership was nonsignificant, *F*_(2, 286)_ = 0.16, *p* = 0.851, η^2^_*p*_ = 0.00; averaging across levels of information, the means in the reason for group membership conditions did not significantly differ.

The main test of *H1* was the reason for group membership X information interaction, which was nonsignificant, *F*_(2, 286)_ = 0.47, *p* = 0.624, η^2^_*p*_ = 0.00 (Table 2). This indicated that the shift in *D* scores in the presence of counterstereotypic individuating information was similar regardless of reason for group membership. Thus, as in the previous studies, *H1* was not supported, and the results from the online sample replicated those from the college sample (Study 2a).

#### Implicit attitudes

As was the case with stereotype *D* scores, we first performed an exploratory ANCOVA on attitude *D* scores with diagnosticity of reason for group membership with regard to trait judgments as covariates. These variables did not covary with *D* scores, *p*s > 0.419.

*H2* was the same as it was in previous studies and thus was tested in the same way. The pattern of results was similar to those from the implicit stereotype measure. There was a significant, large main effect of information, *F*_(1, 281)_ = 48.56, *p* < 0.001, η^2^_*p*_ = 0.15. Averaging across levels of reason for group membership, attitude *D* scores were nonsignificant in the absence of individuating information, *t*_(143)_ = −0.45, *p* = 0.652, and significantly negative in the presence of counterstereotypic individuating information, *t*_(143)_ = −9.54, *p* < 0.001 (Table 1), showing that attitude *D* scores corresponded with the valence of the individuating information. The main effect of reason for group membership was only marginally significant, *F*_(2, 281)_ = 2.91, *p* = 0.056, η^2^_*p*_ = 0.01. Averaging across levels of information, the means in the reason for group membership conditions did not significantly differ.

The main test of *H2* was the reason for group membership X information interaction, which was nonsignificant, *F*_(2, 281)_ = 1.48, *p* = 0.229, η^2^_*p*_ = 0.01. This showed that the shift in *D* scores in the presence of counterstereotypic individuating information was similar regardless of reason for group membership. Thus, as in the previous studies, *H2* was not supported and, as was the case on the implicit stereotype measure, the results from the online sample replicated those from the college sample.

#### Implicit stereotypes controlling for implicit attitudes

To approximate a test of the open research question in the absence of controllability effects, we performed the same analysis as we did in the previous studies. While implicit attitudes significantly covaried with implicit stereotypes, *F*_(1, 270)_ = 96.92, *p* < 0.001, η^2^_*p*_ = 0.26, the pattern of results was unaffected by the addition of this covariate. The information main effect remained significant, *F*_(1, 270)_ = 23.89, *p* < 0.001, η^2^_*p*_ = 0.08 (Supplementary material provide descriptive statistics), showing that *D* scores shifted and became negative in the presence of individuating information even when implicit attitudes were taken into account. This further supported the conclusions reached in the previous studies from this analysis.

The main effect of reason for group membership remained nonsignificant when controlling for implicit attitudes, *F*_(2, 270)_ = 0.50, *p* = 0.608, η^2^_*p*_ = 0.00. The same was true of the reason for group membership X information interaction, *F*_(2, 270)_ = 0.21, *p* = 0.809, η^2^_*p*_ = 0.00.

#### Explicit stereotyping

The same analysis was performed on the explicit stereotype measure in Study 2b as it was in Study 2a. Alpha was adjusted to 0.01 to account for heterogeneity of variances.

The only significant interaction was a significant target X information interaction, *F*_(1, 191)_ = 261.23, *p* < 0.001, η^2^_*p*_ = 0.49, which confirmed that our individuating information manipulation was successful. When only social category information was provided, Mohammad and Rahul were viewed as equally peaceful. However, counterstereotypic individuating information caused Mohammad to be judged as more peaceful than Rahul. Thus, the pattern of results on the explicit measure was similar to that from the IAT except that there was no stereotype effect in the absence of individuating information on the explicit measure.

#### Explicit attitudes

The same analysis was performed on the explicit attitude measure in Study 2b as it was in Study 2a. Alpha was adjusted to 0.01 to account for heterogeneity of variances.

The only significant interaction was a significant target X information interaction, *F*_(1, 275)_ = 101.56, *p* < 0.001, η^2^_*p*_ = 0.27. This further confirmed that our individuating information manipulation was successful. Specifically, when only social category information was provided, participants felt equally warmly toward Mohammad and Rahul. However, when counterstereotypic individuating information was provided, participants felt more warmly toward Mohammad than they did toward Rahul. This corresponded with the valence of the individuating information. This pattern of results replicated that from the implicit measure.

## Study 3a

Study 3a was identical to Study 3b (see below), but used a student sample instead of an online sample. In Study 3a, the individuating information experimental manipulation failed. Therefore, this study is reported in Supplementary material. To address this, we performed Study 4, which was not included in the Stage 1 registered report, but was meant to rectify the problems with Study 3a.

## Study 3b

Although the previous studies tested our hypotheses using existing social groups, they measured existing beliefs about target groups in addition to the processes underlying potential shifts in implicit judgments. Study 3b used novel social groups (the Niffians and the Laapians) as targets to test our hypotheses while removing influences of preexisting beliefs about social groups, thereby enhancing internal validity.

### Method

The procedure and experimental design in Study 3b was largely the same as that in the previous studies, except that the targets and attributes (and thus, the target descriptions) differed.

#### Stimuli and measures

A sample target description from the counterstereotypic information, controllable group membership condition in Study 3b was:

Imagine a group of people called the Niffians. Every member of this group has the letters -nif at the end of their names. The Niffians are usually very competent. They excel academically and in their careers, and they generally enjoy intellectual pursuits such as reading. People choose to join this group; it is not something that group members are born into. Thus, they can leave the group at any time. One particular Niffian, Ibbonif Yossanif Vabbenif, does not quite fit the norm for most Niffians; his defining trait is warmth rather than competence. He cares a lot about others and enjoys socializing.

The other descriptions followed this same template but changed the target groups, individual targets, and attribute used to describe the groups and individual target. The descriptions portrayed the Niffians as competent and their individual member as warm and the Laapians as warm and their individual member as competent.

As discussed above, in Studies 1, 2a, and 2b, stereotypes and attitudes were confounded. In Study 3b, we used similarly-valenced traits (competent and warm) to rectify this. IAT categories were *Ibbonif*, *Reemolap* (the names of the individual novel targets), *competent*, and *warm* (stereotype IAT), and *good* and *bad* (attitude IAT).

#### Participants

After preregistered data exclusions, the final sample size was *N* = 419 Prime Panels workers. The removal of one outlier on the stereotype IAT did not change the pattern of results, so results are reported including this outlier. No participants guessed the purpose of the study.

### Results

#### Implicit stereotyping

We first performed an exploratory ANCOVA identical to that described in Studies 2a and 2b (and in Supplementary material for Study 3a). The diagnosticity variables did not covary with implicit stereotypes, *p*s > 0.262.

To test *H1*, which was the same as in previous studies, the same analysis was performed as in the previous studies. The main effect of information was nonsignificant, *F*_(1, 398)_ = 1.95, *p* = 0.163, η^2^_*p*_ = 0.01; averaging across levels of reason, stereotype *D* scores were inferentially equal in the presence of counterstereotypic individuating information and social category information as they were in the presence of only social category information (Table 1). They were nonsignificant in both conditions, *t*s <1.22, *p*s > 0.113. The main effect of reason for group membership was also nonsignificant, *F*_(2, 398)_ = 0.42, *p* = 0.656, η^2^_*p*_ = 0.00; averaging across levels of information, the means in the reason for group membership conditions did not differ.

The main test of *H1* was the reason for group membership X information interaction, which was nonsignificant, *F*_(2, 398)_ = 0.99, *p* = 0.371, η^2^_*p*_ = 0.01 (Table 2). This indicated that there was no significant shift in *D* scores regardless of reason for group membership. Thus, as in the previous studies, *H1* was not supported.

#### Implicit attitudes

We first performed an exploratory ANCOVA identical to that in the previous studies. The diagnosticity variables did not covary with attitude *D* scores, *p*s > 0.521.

*H2* was the same as in previous studies. Thus, the same analysis was performed to test it. The pattern of results was identical to that from the implicit stereotype measure. The main effect of information was nonsignificant, *F*_(1, 397)_ = 0.88, *p* = 0.350, η^2^_*p*_ = 0.00; averaging across levels of reason for group membership, attitude *D* scores were inferentially equal in the presence of counterstereotypic individuating information and social category information as they were in the presence of only social category information. However, they were nonsignificant in the absence of individuating information, *t*_(197)_ = −0.57, *p* = 0.567, and significantly negative in its presence, *t*_(205)_ = −2.24, *p* = 0.026. The latter result suggested that warmth (and, therefore, Laapians) was evaluated more favorably as a trait than competence (and, therefore, Niffians). The main effect of reason for group membership was nonsignificant, *F*_(2, 397)_ = 1.47, *p* = 0.230, η^2^_*p*_ = 0.01; averaging across levels of information, the means in the reason for group membership conditions did not statistically differ.

The main test of *H2* was the reason for group membership X information interaction, which was nonsignificant, *F*_(2, 397)_ = 0.78, *p* = 0.461, η^2^_*p*_ = 0.00. This showed that there was no significant shift in *D* scores regardless of reason for group membership. Thus, as in the previous studies, *H2* was not supported.

#### Implicit stereotypes controlling for implicit attitudes

As with the previous studies, to approximate a test of the open research question, we performed the same analysis as we did to test *H1* but added implicit attitudes as a covariate. Implicit attitudes significantly covaried with implicit stereotypes, *F*_(1, 381)_ = 15.07, *p* < 0.001, η^2^_*p*_ = 0.04 (see Supplementary material for descriptive statistics). The effect of information remained nonsignificant, *F*_(1, 133)_ = 1.96, *p* = 0.164, η^2^_*p*_ = 0.02, as did the main effect of reason, *F*_(2, 381)_ = 0.32, *p* = 0.726, and the reason for group membership X information interaction, *F*_(2, 381)_ = 1.40, *p* = 0.247, η^2^_*p*_ <0.01.

Covariance of implicit attitudes with implicit stereotypes was not expected in this study (nor in Study 3a) to the extent that it was in Studies 1, 2a, and 2b because the stereotypes that were investigated in this study were intended *not* to involve a valence contrast. What made the stereotypes in Studies 1, 2a, and 2b similar to attitudes was that one target was always characterized by a positive trait and one by a negative trait, but this was not the case in this study. Since both traits were positive, we did not expect differences in attitudes toward the two targets; thus, we expected IAT scores of ~0 for attitudes. While the grand mean attitude *D* score was statistically significant, *M* = −0.04, *SD* = 0.44, 95% CI = (−0.09, 0.00), *t*_(403)_ = −2.00, *p* = 0.046, *d* = −0.10, the *p*-value approached 0.05 and the effect was trivially small; the effect did not reach conventions for a small IAT effect (|*D*| = 0.15; e.g., Rudman, 2011).

#### Explicit stereotyping

##### Individual targets

The same analysis was performed on the explicit stereotype measures in Study 3b as in Studies 2a and 2b. Alpha was adjusted to 0.01 due to heterogeneity of variances.

The only significant interaction in competence ratings for individual targets was a large target X information interaction, *F*_(1, 390)_ = 112.12, *p* < 0.001, η^2^_*p*_ = 0.22, which confirmed that our group trait and individuating information manipulations succeeded. Specifically, when only social category information was provided, participants applied the group stereotypes (i.e., Niffians are competent and Laapians are warm) to the individuals; Ibbonif was judged as more competent than Reemolap. However, when counterstereotypic individuating information was provided that portrayed Ibbonif as warm and Reemolap as competent, Reemolap was judged as more competent than Ibbonif.

For warmth ratings, alpha was adjusted to 0.01 due to heterogeneity of variances. Like the competence ratings, the only significant interaction to emerge was a large, significant target X information interaction, *F*_(1, 390)_ = 198.43, *p* < 0.001, η^2^_*p*_ = 0.34, which confirmed that our group trait and individuating information manipulations were successful. Specifically, when no individuating information was provided, participants applied the group stereotypes (Niffians are competent and Laapians are warm) to the individuals. However, when counterstereotypic individuating information was provided portraying Ibbonif as warm and Reemolap as competent, Ibbonif was judged as more warm than Reemolap.

Thus, results for explicit stereotype-relevant judgments of individuals differed from those of implicit judgments of individuals. The individuating information manipulation was successful at the explicit level but did not affect implicit judgments.

##### Group targets

Here, we focus on main effects relevant to our stereotype induction in addition to the usual focus on reporting the highest-order theoretically meaningful significant effects. For competence ratings, alpha was adjusted to 0.01 due to heterogeneity of variances. Here, the target main effect was significant and large, *F*_(1, 390)_ = 207.23, *p* < 0.001, η^2^_*p*_ = 0.35, which confirmed that our group stereotype induction was successful; participants viewed the Niffians as more competent than the Laapians. The target X information interaction also was significant, *F*_(1, 390)_ = 7.71, *p* = 0.006, η^2^_*p*_ = 0.02. When only social category information was provided, participants judged the Niffians as more competent than the Laapians. When counterstereotypic individuating information was provided portraying Ibbonif as warm and Reemolap as competent, this was still true, but the effect was slightly smaller. This interaction showed that counterstereotypic individual group members slightly weakened the group stereotype.

For warmth ratings, alpha was adjusted to 0.01 due to heterogeneity of variances. In this analysis, the target main effect was significant and large, *F*_(1, 390)_ = 231.82, *p* < 0.001, η^2^_*p*_ = 0.37, and confirmed that our group stereotype induction was successful; participants viewed the Laapians as warmer than the Niffians. No other effects were significant.^10^

#### Explicit attitudes

Because we utilized two positive traits in this study, we did not expect attitude effects. Nonetheless, several significant effects emerged. These are reported in Supplementary material because they are relevant to the perceived favorability of warmth vs. competence rather than to our research questions.

## Study 4

Study 4 was conducted upon speculating that the reason for Study 3a's individuating information manipulation failure was that the study was too cognitively taxing for the population utilized in that study, as evidenced by high rates of manipulation check failures (30.69%). Study 4 was not included in the Stage 1 registered report. Study 4 was designed to simplify Studies 3a and 3b; the information that participants needed to recall was half of that required in the previous studies (see below). Although in the registered report we stated that we would investigate the question of controllability and individuating information effects using novel group targets among college students, since the manipulation failed in that population, we decided to conduct this study using Prime Panels. We saw no disadvantage to this because online samples tend to be more representative than college samples (for a review, see Gosling and Mason, 2015).

### Method

The method was identical to that in the previous studies with two exceptions. The first was that participants saw only one target description (highly similar to one from Study 3a and 3b) and all subsequent measures related only to that target. Because of this, the within-subjects target factor was not present in the experimental design. The design was otherwise identical to that from the previous studies. The single target description portrayed the Niffians as competent and, when counterstereotypic individuating information was provided, the individual target, Ibbonif, as warm and not competent.

The second change was that the Single-Target Implicit Association Test (ST-IAT; Wigboldus et al., 2004) was used instead of the IAT. The logic behind the ST-IAT is identical to that for the IAT. The differences are that only one target group or individual is used, and that there are therefore five blocks of trials instead of seven.

#### Participants

A power analysis using an effect size of *f* = 0.15, 80% power, and alpha of 0.05 showed that 432 participants were needed. After using the same data exclusion criteria as in the previous studies, the final sample size was *N* = 506 Prime Panels workers. Despite our efforts to simplify the study, there was a high rate of manipulation check failures (24.02%). The removal of ST-IAT score outliers did not affect the results, so results are reported including these outliers. No participants guessed the purpose of the study.

#### Results

In this study, positive stereotype *D* scores showed that participants associated Ibbonif with competence (the group stereotype) more than warmth, and negative *D* scores showed that the reverse was true (i.e., that Ibbonif was judged according to the individuating information). Positive attitude *D* scores showed that participants associated Ibbonif with *good* more than *bad*.

#### Implicit stereotyping

We first performed an exploratory ANCOVA identical to that described in the previous studies. The diagnosticity variable did not covary with implicit stereotypes, *p* = 0.839.

To test *H1*, which was the same as in previous studies, the same analysis was performed as in the previous studies. The main effect of information was significant, *F*_(1, 483)_ = 37.49, *p* < 0.001, η^2^_*p*_ = 0.07. However, the means were in an unexpected direction; *D* scores were significantly *higher* in the presence of counterstereotypic individuating information and social category information, single-sample *t*_(237)_ = 6.84, *p* < 0.001, than they were in the presence of only social category information, single-sample *t*_(251)_ = −1.88, *p* = 0.061 (Table 1), revealing that participants judged the target to be *more* consistent with the group stereotype when counterstereotypic information was provided. However, the magnitude of *D, M* = 0.10, was significantly below the threshold for a small IAT effect (|*D*| = 0.15; Rudman, 2011), *t*_(237)_ = −2.97, *p* = 0.003, *d* = 0.19, even if alpha is conservatively adjusted to 0.01. Thus, though statistically significant, the unexpected direction of the judgment was trivial in magnitude. The main effect of reason for group membership was nonsignificant, *F*_(2, 483)_ = 0.86, *p* = 0.425, η^2^_*p*_ = 0.00; averaging across levels of information, the means in the reason for group membership conditions did not differ.

The main test of *H1* was the reason for group membership X information interaction, which was nonsignificant, *F*_(2, 483)_ = 0.40, *p* = 0.669, η^2^_*p*_ = 0.00 (Table 2). This indicated that there was no shift in *D* scores regardless of reason for group membership. Thus, as in the previous studies, *H1* was not supported.

#### Implicit attitudes

We first performed an exploratory ANCOVA identical to that in the previous studies. The diagnosticity variable did not covary with attitude *D* scores, *p* = 0.905.

*H2* was the same as in previous studies. Thus, the same analysis was performed to test it. The main effect of information was nonsignificant, *F*_(1, 500)_ = 1.12, *p* = 0.291, η^2^_*p*_ = 0.00. Averaging across levels of reason for group membership, attitude *D* scores were inferentially equal in the presence of counterstereotypic individuating information and social category information as they were in the presence of only social category information. However, *D* scores in the former condition were nonsignificant, *t*_(245)_ = 6.84, *p* = 0.209, while those in the latter condition were positive and significant, *t*_(260)_ = 2.95, *p* = 0.004. The main effect of reason for group membership was only marginally significant, *F*_(2, 500)_ = 2.52, *p* = 0.082, η^2^_*p*_ = 0.01; averaging across levels of information, the means in the reason for group membership conditions did not statistically differ.

The main test of *H2* was the reason for group membership X information interaction, which was nonsignificant, *F*_(2, 500)_ = 0.90, *p* = 0.405, η^2^_*p*_ = 0.00. This showed that there was no shift in *D* scores regardless of reason for group membership. Thus, as in the previous studies, *H2* was not supported.

#### Implicit stereotypes controlling for implicit attitudes

As in the previous studies, to approximate a test of the open research question, we performed the same analysis as we did to test *H1* but added implicit attitudes as a covariate. Implicit attitudes did not significantly covary with implicit stereotypes, *F*_(1, 482)_ = 1.21, *p* = 0.271, η^2^_*p*_ = 0.00 (see Supplementary material for descriptive statistics). The effect of information remained significant, *F*_(1, 482)_ = 38.02, *p* < 0.001, η^2^_*p*_ = 0.07, and the main effect of reason remained nonsignificant, *F*_(2, 482)_ = 1.00, *p* = 0.368. The reason for group membership X information interaction also remained nonsignificant, *F*_(2, 482)_ = 0.36, *p* = 0.699, η^2^_*p*_ = 0.00. The covariance of implicit attitudes with implicit stereotypes was not expected in this study to the extent that it was in Studies 1 and 2 for the same reasons as in Study 3b.

#### Explicit stereotyping

##### Individual target

Since there was only one individual target in this study (Ibbonif), we performed 3 (reason for group membership: no reason vs. controllable reason vs. uncontrollable reason) × 2 (information: social category information only vs. social category and individuating information) between-subjects ANOVAs. Alpha was adjusted to 0.01 due to heterogeneity of variances.

Competence results for individual targets are reported in Supplementary material because warmth ratings were most relevant to the individuating information effect. A significant, large information effect, *F*_(1, 482)_ = 185.07, *p* < 0.001, η^2^_*p*_ = 0.28, showed that Ibbonif was rated as warmer in the social category and individuating information condition than in the social category information condition. This provided the most direct evidence that our individuating information manipulation (portraying Ibbonif as warm but not competent) was successful. Thus, as in Study 3a, the results from the explicit stereotype measure for individual targets diverged from those of the implicit measure.

##### Group target

It should be noted that unlike Study 3a, the ANOVAs for the group target were irrelevant to the success of the experimental manipulation; because there was not a second target group, there was no target main effect that could have emerged. Thus, they are reported in Supplementary material. A single-sample *t*-test comparing the grand mean to the midpoint of the competence scale was the test of the success of our group stereotype induction because in all conditions (regardless of whether individuating information was provided), we would expect the Niffians to be rated as highly competent. This test confirmed the success of our group stereotype induction (Supplementary material).

#### Explicit attitudes

These analyses are reported in Supplementary material for the same reasons as in Study 3b.

## General discussion

We predicted that reductions in implicit stereotypes and prejudices would be less pronounced when group membership was portrayed as controllable than when it was portrayed as uncontrollable or than when no reason for group membership was provided. Across six studies, we found a relatively consistent pattern of results. When targets were members of existing social groups, *D* scores shifted and became negative in the presence of counterstereotypic individuating information, and controllability of group membership did not significantly influence this. When targets were members of novel social groups, individuating information either did not influence implicit evaluations and stereotype-relevant judgments of group members or did so in an unexpected (but trivially small) direction, and controllability of group membership did not affect this. Thus, none of the key hypotheses proposing that controllability of group membership should moderate reliance on social category information and individuating information were supported. In addition, when stereotype-relevant implicit judgments involved a valence contrast, implicit attitudes accounted for separate variance in the data, revealing that implicit stereotypes and attitudes were not entirely redundant despite valence contrasts. Finally, results from explicit measures mirrored those from implicit measures when targets were members of existing, but not novel, social groups.

### Differences between existing and novel social groups

It was surprising that there was either no effect of individuating information on implicit judgments of individual members of novel social groups, or that the effect was in the opposite direction as we predicted. This may have occurred because the studies were cognitively taxing for participants; high rates of failures of manipulation checks–even in the study designed to be less cognitively taxing–were evidence of this. Even when participants passed these manipulation checks and were retained in the dataset, it is possible that some were guessing when answering these questions given that these questions were in multiple choice format. The explicit stereotype measures for Study 3a (reported in Supplementary material) also showed that, although a group stereotype formed, targets were not individuated even at the explicit level. This is further evidence for the notion that the studies were too cognitively taxing for participants because individuating information effects are generally quite strong at the explicit level (one meta-analysis showed an average effect size of *r* = 0.71; Kunda and Thagard, 1996); a complete lack of such effects is rare and surprising.

In addition, although previous research has had success using the novel groups paradigm in the context of implicit attitudes (e.g., Kurdi and Banaji, 2017), the design of our study is more cognitively taxing than previous ones. This is because, in our study, participants had to learn (a) a stereotype of one or more novel groups; (b) half the time, information about one or more specific members of the group that contradicted the stereotype; and (c) for most participants, reasons for group membership. In previous research, participants only learned attributes of the group and applied them to its individual members.

### Lack of controllability effects

Most previous research has found effects of controllability on biases against individuals and groups; ours did not, either at the implicit or explicit level. One possible explanation for this is that previous research did not test controllability as it pertained to the effects of *individuating information* on social category effects, as we did. Individuating information effects are far larger than social category effects—*r* = 0.71 vs. *r* = 0.25, respectively, according to Kunda and Thagard's (1996) meta-analysis. While the present research examined social category effects like previous research finding controllability effects did, it investigated how strong such effects were *in the presence of individuating information*. It is possible that individuating information effects are so strong, especially as compared to social category effects, that they are not as malleable as the social category effects that have been found to be influenced by controllability. Thus, individuating information effects may continue to exert influence on judgments regardless of controllability of group membership.

Indeed, the finding that perceived controllability of group membership did not moderate the effects of individuating information on implicit beliefs and attitudes in implicit person perception is consistent with some previous research which found that individual differences known to exacerbate stereotypes and prejudice (e.g., high dogmatism) did not diminish individuating information effects in implicit stereotype-relevant judgments of individuals (Rubinstein et al., 2023). Like the present research, that research found that individuating information effects were consistent despite the presence of factors known to exacerbate biases, which one would expect to reduce individuating information effects.

### Contributions and implications of the present research

Despite the lack of support for our hypotheses, the present research does contribute to understanding stereotypes and attitudes in implicit person perception. It is the first research of which we are aware to investigate whether counterstereotypic individuating information shifts *D* scores measuring biases against individual targets belonging to weight groups (see McConnell et al., 2008 for the effects of *counterattitudinal* information on implicit judgments of such groups) and religious groups. Indeed, both attitude and stereotype *D* scores significantly shifted and became negative in the presence of counterstereotypic individuating information for both types of targets. Thus, the present research generalizes previous findings that addressed this question in the domain of race and gender groups (e.g., Cao and Banaji, 2016; Rubinstein et al., 2018).

In addition, we hypothesized that individuating information would reduce social category effects less when group membership was portrayed as controllable compared to when group membership was portrayed as uncontrollable or when no reason for group membership was provided. The fact that individuating information influenced social category effects equally robustly regardless of controllability of group membership underscores how promising a means of bias reduction individuation can be; despite a factor (controllability) found in past literature to amplify biases, individuation was an effective means of robustly shifting *D* scores in the negative direction in implicit judgments of members of known social groups.

Moreover, although the present research was not meant to test theories of the processes underlying implicit social cognition, the studies on existing social groups did lend indirect support to propositional, fast-learning accounts of implicit evaluations (e.g., De Houwer, 2014) and those predicting fast-learning and the influence of propositional processing on associative processes in the evaluative circumstances posed by the present research (e.g., Gawronski and Bodenhausen, 2006, 2011). Specifically, the information presented to participants was propositional in nature, and participants' implicit evaluations and stereotypes of members of existing social groups were rapidly updated to be in accordance with the propositional information that they learned.

Finally, we found consistent evidence that implicit attitudes did not account for the effects of individuating information on implicit stereotypes. This supports the notion that, among the stereotypes studied in Studies 1, 2a, and 2b, attitudes and stereotypes are at least somewhat separate processes (despite their covariance). This stands in contrast to several other stereotypes involving valence contrasts (Kurdi et al., 2019).

### Limitations and future directions

One limitation of the present research was that it tested the proposed effects among only two types of extant social categories: those based on weight and those based on religion. It is possible that controllability effects would have emerged if additional types of target groups had been studied. Future research should address this question.

Moreover, in Studies 3a, 3b, and 4, participants failed manipulation checks at a high rate. It is possible that the study was too challenging for the participant populations (especially the student population utilized in Study 3a, where the individuating information manipulation failed). Future research might be able to avoid the null results found in these studies by further simplifying the information presented.

Finally, the undergraduate samples utilized in the present research were drawn from a liberal, northeastern population. Although this limitation was in part addressed by replicating the results using online samples, future research might use online samples where participants are selected based on census-matched strata.

## Conclusion

The present research was the first of which we are aware to test the question of whether perceived controllability of social group membership reduces the impact of individuating information on social category effects in implicit person perception. Across multiple existing social categories, it did not. This suggests that individuation is a particularly robust and promising means of implicit bias reduction in that circumstances known to exacerbate biases did not reduce individuation effects in the present research.

## Data availability statement

The datasets presented in this study can be found in online repositories. The names of the repository/repositories and accession number(s) can be found in the article/supplementary material. Supplementary material and preregistrations for all studies can be found at https://osf.io/hw7ar/?view_only=baab178ea4444038b8376ee0db460245. Studies 3a and 3b were not preregistered on OSF due to an oversight, but were preregistered in that the research was in the registered report format.

## Ethics statement

Studies 1, 2a and 3a were approved by both the Towson University Institutional Review Board and the Rutgers University Institutional Review Board. Studies 2b, 3b, and 4 were approved by the Towson University Institutional Review Board. Written informed consent to participate in this study was provided by the patient/participants or patient/participants' legal guardians/next of kin.

## Author contributions

RR and LJ designed the studies. RR analyzed the data and drafted the manuscript. LJ, BM, KS, SY, and SB revised the manuscript. All authors assisted with data collection. All authors contributed to the article and approved the submitted version.

## Footnotes

^1^To truly measure diagnosticity of the *reason* for the targets' weight with regard to judgments of motivation/laziness, which was our intention, the question should have been “To what extent did the *reason* for [target's] weight inform your judgment of how motivated or lazy he is?” We changed this question accordingly in subsequent studies. We did not analyze data from this measure in Study 1 because it did not address the question that we intended it to. However, this question was irrelevant to our hypotheses.

^2^This question was revised slightly from the version proposed in the Stage 1 submission to improve clarity and change the response scale from a unipolar scale to a bipolar scale.

^3^We also attempted multiple transformations of the IAT data in Study 2b to correct the issue (log transformations and square root transformations, both after adding a constant to change the lowest value in the dataset to be 1). This did not resolve the issue. Keppel and Wickens (2004) suggested dividing alpha by two as an alternative, so we chose to be more conservative than that and set alpha to 0.01. Since transformations were not successful at fixing the issues with our main dependent measure—IAT scores—we chose to utilize this approach for all measures showing heterogeneity of variances for consistency.

^4^Although the experimental design described earlier included a within-subjects target factor, this preregistered analysis (and an identical one using attitude *D* scores as the dependent variable) was performed because, since IAT scores are difference scores involving judgments of the two targets, they inherently incorporate the within-subjects target factor. The same is true for the remaining studies.

^5^IAT order (attitude IAT first vs. stereotype IAT first) did not affect the main tests of the hypotheses in any of the six studies as tested by the IAT order X information X reason for group membership interactions (*p*s > 0.372) and these interactions also were nonsignificant excluding IAT order in all studies. Therefore, analyses are reported in all studies excluding this factor (see Supplementary material for results of these analyses for all studies).

^6^Effect sizes for single-sample *t*-tests on *D* scores are not reported in this manuscript because the computation of *D* closely resembles the computation of Cohen's *d*.

^7^Secondary analysis revealed that perceived credibility of individuating information also did not significantly covary with stereotype *D* scores in any of the six studies, *p*s > 0.190 (see Supplementary material for these results for all studies).

^8^Secondary analyses revealed that perceived credibility of individuating information also did not significantly covary with attitude *D* scores in any of the six studies, *p*s > 0.083 (see Supplementary material for these results from all studies).

^9^This was not preregistered, but we felt it was necessary because implicit preferences for ingroup members oftentimes differ from those for outgroup members (e.g., Lai and Banaji, 2020).

^10^Explicit and implicit results for group targets in Studies 3b and 4 are not compared because the IATs in those studies only pertained to individual targets.
